# Investigating Reflexive Responses to Explicit and Implicit Forms of Social Exclusion Using Immersive Virtual Environment Technology

**DOI:** 10.3389/fpsyg.2020.575783

**Published:** 2020-10-06

**Authors:** Claire Nicole Prendergast, Thomas Schubert

**Affiliations:** Department of Psychology, Faculty of Social Sciences, University of Oslo, Oslo, Norway

**Keywords:** forms of social exclusion, immersive virtual environments (IVE), reflexive responses to social exclusion, negative affect, EDA (electro dermal activity), HRV (heart rate variability)

## Abstract

Humans are strongly affected by social exclusion, a multifaceted and complex phenomenon of social life. However, individuals tend to respond differently depending on a multitude of individual and contextual factors. Firstly, with a view to increasing the ecological validity and experimental control of an exclusion manipulation in the laboratory setting, we made use of immersive virtual environment technology (IVET; an Oculus Rift Virtual Reality headset) to create a new exclusion paradigm. Secondly, given that a recent meta-analytic report on reflexive responses (i.e., affect and physiology) to manipulations of exclusion in the laboratory setting cites inconsistencies across findings (Blackhart et al., 2009), we focused on the form of exclusion manipulated to illustrate how this factor may help to explain the divergences in responses. We thus investigated how explicit and implicit forms of social exclusion may have a differential impact on self-reported affect, as well as on electrodermal and cardiovascular activity. Results from this laboratory study conducted with a varied sample of the local population made salient the affordances of IVET as a tool in exclusion research. They also helped to reconcile the conflicting findings in the literature relating to differences in the level of negative affect generated and shed light on physiological arousal in the wake of being excluded in different ways.

## Introduction

Social exclusion is ubiquitous to human experience. But individuals and groups can be socially excluded in many different ways and contexts throughout a lifetime. Whether, as a child, the schoolyard bully tells us that we cannot play with the group, or as an adult, we are left out of a work event, one thing we know is that the hurt runs deep. How we respond to these different events, however, may differ greatly according to just how explicit and direct, or implicit and indirect, the exclusionary event is. Much experimental research on social exclusion to date has documented the effects that the threat or experience of social exclusion as a *generalized* phenomenon can have on the mind and body (Dewall et al., 2009; Smart Richman and Leary, 2009; Richman, 2013; Stenseng et al., 2015; Beekman et al., 2016). As such, researchers commonly use experimental manipulations of exclusion such as the *alone-later-in-life* paradigm (Twenge et al., 2002) or the *cyberball* paradigm (Williams and Jarvis, 2006). However, these paradigms often do not take into account the form of exclusion experienced by participants, i.e., how explicit and intentional, or implicit and unintentional the event may have been. Cyberball, as it is generally manipulated, can be ambiguous in its implementation leaving participants wondering if the other ball throwers actually meant to exclude them or if it was just an oversight.

While much of this type of generalized research on exclusion has been informative, and indeed successful in rendering feelings of exclusion in study participants, findings are limited in a number of ways, both generally with respect to experimental research and specifically, in the case of manipulations of exclusion. These limitations include lack of real-world application and reduced experimental control (i.e., a rarely experienced social event or using a laboratory setting with live actors), low ecological validity that is associated with proxy paradigms (e.g., operating and interacting with avatars or agents online, such as in *cyberball*) and finally, lack of specificity (i.e., the form or extent of the exclusion experienced is not a focus of the research).

In the present study, we had both a methodological and conceptual aim. Our methodological aim was to improve the experimental control and ecological validity of exclusion manipulations in the laboratory. To this end, we developed a new exclusion paradigm using immersive virtual environment technology (IVET) whereby participants wore a Virtual Reality headset to experience exclusion in a social group through the first-person perspective. Our immersive virtual environment (IVE) was thus a VR-rendered 360° film of a real-life social group interacting in a natural social setting and eventually enacting an exclusion on the participant. This exclusion occurred with respect to a commonly experienced social event (i.e., meeting new colleagues on the first day of a new job in the workplace canteen). The novel method used in this experiment thus eliminated the variation that would otherwise be introduced by live actors in the laboratory. Moreover, it mitigated the impact of the laboratory setting on results, the so-called Hawthorne effect and finally, it removed the proxy effect of avatars or agents on results.

In addition, our conceptual aim was to increase the specificity of the exclusion event with a view to reconciling the conflicting findings reported in a metanalytic review of hundreds of exclusion studies by Blackhart et al. (2009). We posit that what is missing from many empirical studies on exclusion is a focus on form, i.e., how explicit or implicit the exclusion is, that may help to explain different patterns in responses. As such, we manipulated two types of exclusion; characterized as explicit and direct, and implicit and indirect. We hypothesized significant differences across affective and physiological responses to these two different forms of exclusion and interpreted results in light of the existing literature (Blackhart et al., 2009). Our dependent variables were therefore related to self-report positive and negative affect (NA) as well as electrodermal responses and cardiovascular activity.

Finally, to further elucidate the differential impact of explicit and implicit forms of exclusion on responses and contextualize the main affective and physiological responses to both forms, we included measures for individual traits and prior relational experiences that are generally implicated in conditions of exclusion. As such, we measured vulnerable narcissism, attachment style, loneliness and previous experiences of discrimination and rejection two weeks prior to the laboratory experiment. In general, narcissism has been linked to aggressive behavior following exclusion (Twenge and Campbell, 2003) and attachment styles have been shown to moderate responses to ostracism (Shaver and Mikulincer, 2013). Similarly, loneliness and prior experiences of discrimination and rejection are thought to impact responses to new or ongoing experiences of exclusion (Smart Richman and Leary, 2009; Hawkley et al., 2013). We predicted that these relational variables would be primarily linked to responses to implicit exclusion and that, responses to explicit exclusion would be primarily explained by the directness of the exclusionary event in and of itself.

### Immersive Virtual Environment Technology

**FIGURE 1:**
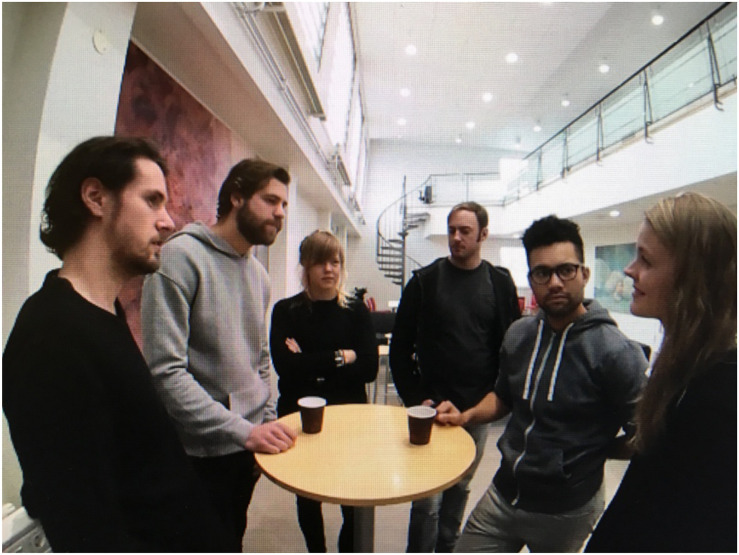
First-person perspective of the group as experienced by participants in the IVE.

Though not exactly new, IVET presents psychologists with an opportunity to achieve higher ecological validity in the experimental setting, and to our knowledge, has yet to be used in the context of social exclusion research in this way. Some existing reports employ the term Immersive Virtual Environments (IVE) to refer to online social worlds in which exclusion can be manipulated via *cyberball* interactions (Kassner et al., 2012). However, in these studies, participants sat in front of computer screens and were not virtually “immersed” in the scene itself, as is now possible with the use of Virtual Reality headsets. These previous reports therefore focused on issues of agency in relation to avatars and agents. In our case, however, an Oculus Rift VR headset was used. Participants thus experienced the exact same experimental manipulation of either explicit or implicit exclusion from a social group through the first-person perspective, as though they were actually standing in the group (see Figure 1 for this perspective). As such, in IVET terms, *immersion* is an objective description of the technical capabilities of the Virtual Reality system, that describes the level of detail with which a virtual environment can be rendered (Blascovich et al., 2002). Furthermore, *presence* describes the user’s psychological response to said environment (Schubert et al., 2001; Blascovich et al., 2002).

The methodological offering of the present study is such that we expected *behavioral realism* and thus *immersion* to be higher than other studies given the high resolution of the 360° film and playback in the Oculus Rift VR headset. On the other hand we envisaged that *presence* would be slightly lower than a real-life social experience of exclusion, given that our stimuli were pre-recorded 360° videos of a social group and thus, non-interactive (though some participants did try to interact). Some level of imagination is still required of participants in order to engage with this type of stimuli, so while we kept the social scenario in the IVE as close to a commonly shared real-life experience as possible (i.e., meeting new colleagues on the first day of a new job), we made sure to control for its effectiveness by implementing multiple manipulation checks that helped to validate this novel method. The study was promoted as being about “social life” in general and participants had no way of knowing that they would experience social exclusion.

### Reflexive Responses to Social Exclusion

The present study focused on reflexive responses to exclusion that have been previously modeled as the most immediate ways in which individuals react to being excluded (Hawkley et al., 2011). We therefore assume that they are regulated at the level of neurobiology and constitute affective and physiological responses that are amenable to measurement in the experimental setting. These responses provide a crucial and – in the case of physiological data – unbiased first indication of how individuals have been impacted by the exclusionary event.

Affective responses are commonly measured through the self-report Positive and Negative Affect Scale (PANAS; Watson et al., 1988). Physiological responses are usually tapped through electrodermal activity (EDA) and, in our case, plethysmography (PPG), the latter from which we calculated pulse rate (PR) and pulse-rate variability (PRV), before, during and after participants had been excluded in the experiment. EDA provides an indication of eccrine activity (skin sweating) that is linked to psychophysiological arousal due to sympathetic neuronal activity. Data is generally analyzed with respect to skin conductance levels (SCLs), which is the tonic signal that changes slowly across time and, skin conductance responses (SCRs), which are derived from the phasic signal and constitute a measure of individuals’ event related responses (ER-SCRs) or non-specific galvanic skin conductance responses (NS-SCRs) (i.e., sharp responses indexed by amplitude, latency, and recovery periods) during experimental manipulations (Boucsein, 2012). PR and PRV can be modeled as surrogates for heart-rate (HR) and heart-rate variability (HRV), respectively. PR data collected using PPG has been aligned with HR data collected using ECG under conditions of physical inertia and, as such, is believed to constitute a reliable proxy measure of heart rate and heart rate variability (Lin et al., 2014).

Reflexive responses are therefore modeled herein as a broad category and while they are commonly attributed to the need-threat model (Williams and Zadro, 2001) in exclusion research, we have instead opted to apply a focus on the form of exclusion experienced (i.e., explicit or implicit) in the present study, to show how this feature alone can go a long way in accounting for differing patterns in affect, electrodermal and cardiovascular activity subsequent to the experience of each form of exclusion.

### A Focus on Form of Exclusion

While much research on social exclusion, when treated as a generalized phenomenon, has been informative, there has been a vast array of differing and often opposing responses recorded in the experimental setting that we show may be primarily due to the lack of specification of the exclusionary event and how this is modeled in the data.

#### Affective Responses

In a meta-analytic review of 192 studies using *cyberball* and *alone-later-in-life* paradigms, Blackhart et al. (2009) outlined numerous inconsistencies in the reflexive responses recorded across studies. Crucially, this report showed that in some studies with respect to self-report positive and NA, an emotionally neutral state is triggered by the experience of social exclusion, while in others, a significant increase in NA is reported. There are indeed theoretical explanations for both responses, but no account is provided in the report as to why the findings differ so greatly across studies and what may determine one response over another. While there may be some statistical limitations in the presentation of this metadata, we posit that the inconsistencies may be largely explained by a lack of focus on the form of exclusion experienced by participants in these studies.

In the standard implementation of the *cyberball* paradigm, for instance, some participants may experience the exclusion as explicit and direct (e.g., the other players actively sought to exclude the participant), while others may see it as more implicit and indirect (e.g., the other players may have forgotten about the participant), and therefore, the form of exclusion is ambiguous. This ambiguity makes for increased noise in the data tracking responses. Moreover, in an fMRI study that did specify the form of exclusion by implementing both an explicit and implicit form of cyberball, results pointed to the activation of different neural correlates for each form, as well as differing patterns within the dorsal area of the brain (Eisenberger et al., 2003). However, no discussion of this outcome was included in the report. Blackhart et al. (2009) alluded to this feature in reviewing the metadata also, suggesting that it is “the extent of the exclusion” experienced that may explain the divergent responses found in the literature, from explicit to implicit across a continuum.

Other research has suggested that characterizing exclusion in terms of its form (i.e., explicit or implicit) is an important step in understanding and explaining how individuals are differentially affected (Molden et al., 2009). To help characterize exclusionary events as such, Molden et al. (2009) theorize that explicit, and thus direct and overt acts of exclusion imply a social loss while implicit forms, that are more passive and covert, entail a failure for social gain. For instance, being refused entry to a nightclub by a doorman (an explicit exclusion) prevents the individual from entering into that social arena against their will. By contrast, feeling like one does not fit in at the local gym in the presence of more experienced gym users (an implicit exclusion) will likely lead to the individual’s withdrawal from the social arena by choice. Acts of explicit exclusion are intentional, external and usually irreversible, hence the social loss. Acts of implicit exclusion can be intentional (e.g., being ignored when speaking in a group) or unintentional (e.g., the individual feels they do not fit in), but nevertheless hinge more on the target individual’s interpretation of the event as exclusionary, an internal evaluation that implicates, among many, the relational variables and prior experiences of exclusion that we aimed to measure herein.

Other proposals have been made to model exclusion events according to form. Indeed, simultaneous to our own research, Wesselmann et al. (2019) called for a comparison between different types of exclusion to shed light on the array of responses that each form may differentially elicit. To the best of our knowledge, no other empirical work to date has tested responses to explicit and implicit forms of exclusion in this way.

Finally, we hypothesized that the strength of responses to explicit forms of exclusion would be explained by the exclusionary act in and of itself due to the undeniable intentionality and finality of the event and that, conversely, responses to implicit exclusion would unfold as a function of individual differences in key related variables and prior experiences of exclusionary events.

#### Physiological Responses

Inconclusive and conflicting reports also permeate the literature investigating physiological responses to cyberball manipulations, to the extent that the experience of social exclusion itself has been thought not to uniformly impact physiological activity (Williamson et al., 2018). Similar to the divergences that have been recorded in affective responses to exclusion (Blackhart et al., 2009), previous research has not been successful in finding conclusive differences in EDA using cyberball manipulations of exclusion and acceptance (e.g., Iffland et al., 2014). In addition, some studies assessing changes in cardiovascular activity seem to suggest that HR should increase during cyberball manipulations of exclusion and decrease during acceptance manipulations (Iffland et al., 2014), while other findings, using alternative manipulations, have pointed to a deceleration of HR during the experience of exclusion, a so-called “freezing” effect, but that the recovery to baseline is somewhat predictable, presumably affecting HRV results overall (Papousek et al., 2014). HRV is known to decrease during stressful situations in general, and this effect has also been found to occur in response to exclusion (Pieritz et al., 2017).

### The Present Study

While numerous studies have looked at affective and physiological responses to cyberball manipulations of acceptance and exclusion (Iffland et al., 2014; Kouchaki and Wareham, 2015; Durlik and Tsakiris, 2015; Williamson et al., 2018), none have compared responses across two distinct forms of social exclusion (i.e., explicit and implicit forms) in a social setting high in behavioral realism, such as the present study. Accordingly, the aim of the current study was to provide an analysis of differences that may occur in response to both forms of exclusion. As such, we investigated self-report positive and NA as a function of explicit or implicit exclusion. We also explored EDA changes in SCL during the experimental manipulation carved up into three segments: pre-, during and post-exclusion and contrasted the latter two segments with two baseline measures of the participants’ SCL taken from sequences in empty IVE rooms before the experimental manipulation took place. In addition, we compared ER-SCRs from the explicit exclusion condition with NS-SCRs in the implicit exclusion condition and examined the amplitude and frequency of both measures across conditions. Similarly, from the two baseline readings in the empty IVE rooms, we compared changes in HR and HRV across the same three segments of the experimental manipulation: pre-, during and post- exclusion using difference scores.

#### Experimental Control and Hypotheses Testing

In the present study, using an Oculus Rift VR headset meant that every participant experienced the exact same exclusionary event rendered in a more real life setting (e.g., a workplace canteen) through the first-person perspective. This removed noise that would otherwise be associated with the variation in live exclusion manipulations played by actors in a laboratory setting and indeed, the “proxy” effect of avatars in online games, working toward our methodological aim which was in increasing the ecological validity of the event with respect to these parameters.

Our conceptual aim was in drawing comparisons between reflexive responses to each form of exclusion, explicit or implicit, to shed light on the inconsistencies cited in the literature (Blackhart et al., 2009). The empirical focus of the experiment was therefore on the form of social exclusion experienced as assumed and not, for example, the outcome of being excluded *in general*, which is commonly contrasted with the outcome of being socially accepted. As such, a control condition was not deemed necessary to achieve our aims. Rather, experimental control was fulfilled through numerous follow-up measures and manipulation checks, including a one-item measure of *perceived level of exclusion or inclusion* from the social group.

Furthermore, we sought to test and control for two additional factors; “desire to join the group” and “motivation to join the group.” In one condition, the group espoused progressive opinions on the topic under discussion and in a second, they upheld more traditional ideals. As we had pre-recorded participants’ own views on the topic, we modeled this aspect as their “desire to join the group” given their shared values with either group. We also gave participants an intrinsic motivation to join the group (simply try to make a good impression in the group) or an extrinsic motivation (make a good impression for future promotion in the workplace). These factors did not impact the results significantly and since they were not crucial to the main hypotheses, they acted as controls for the study content and are further documented in the Supplementary Material only.

Finally, our main hypotheses were as follows. First and foremost, we predicted simply that the experience of explicit exclusion would lead to *stronger* affective, electrodermal and cardiovascular responses than implicit exclusion, for example, by generating higher levels of NA, higher SCLs and lower HRV. Secondly and more generally, we expected that the reflexive responses of those who experienced implicit exclusion would be correlated significantly with the other relational traits we measured such as narcissism and insecure attachment styles, as well as prior experiences of exclusion and discrimination and that conversely, these correlations would not be present when accounting for the reflexive responses of those in the explicit exclusion condition.

## Materials and Methods

### Exclusion Criteria

The target sample was age-restricted due to the relevance of the study materials. The social group who appear in the IVE are described to participants as employees of a “young creative company.” Therefore, the target age range for the sample was set at 18–40 years old. We set our exclusion criteria as the following: significant medical or neurological illness; history of substance misuse (drugs/alcohol); history of psychosis or other psychological disorders; significant suicidal ideation or behavior (during the last 6 months); visual impairment - including strabismus, previous ocular surgery, corneal irregularity, opacification of ocular media including cataracts or active ocular disease; frequent dizziness, vertigo and imbalance; previous diagnosis of photosensitive epilepsy. As such, individuals who ticked any of the exclusion criteria in the pre-screen survey were not contacted to take part in the laboratory study. Those who were eligible were contacted by phone or email and invited to come into the laboratory to participate.

Five individuals who completed the experiment were excluded from the study during the testing period. Two of these were excluded because they knew one or more of the actors in real life. One was excluded due to non-native proficiency in the Norwegian language and the final two participants were excluded due to behaviors that signaled lack of comprehension of the study materials and instructions during the experiment (e.g., did not look around in the IVE, did not properly read the instruction sheet etc.).

### Participants

Participants were primarily recruited using Facebook and Instagram advertising. The target audience of the advertisement was individuals aged between 18 and 40 years old and resident in Oslo, Norway. About 100 flyers were randomly handed out in public spaces in central Oslo, together with about 20 notices that were posted on bulletin boards in various city center locations. Participants were also recruited within the student population of the University of Oslo through an online Research Pool using Sona Systems© (Sona, 2018), that offers course credit in exchange for participation in research projects.

Individuals were first invited to fill out an online survey (created using Qualtrics, 2018) hosted at a URL with a view to participating in a laboratory study on social life using virtual reality technology. A 200 NOK (∼$25) universal gift card was offered as a reward for eventual participation in the laboratory study.

### Ethics

The study content and data management protocol were approved by the national data protection service in Norway and the Institutional Review Board. Participants gave their explicit consent for participation in the pre-screen survey online by signing their names in a consent box and provided their details to be contacted for participation in the laboratory study.

Individuals who were invited to take part in the laboratory study were asked to carefully read and sign an informed consent form at the laboratory before participating in the experiment. They were also fully debriefed after the experiment and asked to sign a non-disclosure information sheet. They were presented with a 200 NOK (∼$25) universal gift card at the end of the experiment. All participants were given the opportunity to ask questions or retract their participation during the study, and up to 3 months after the study had been completed.

Written informed consent was obtained from the actors for the publication of any potentially identifiable images or data included in this article.

### Sample Size and Power

In a meta-analytic study comprising 120 cyberball studies comparing responses to experimental manipulations of acceptance and exclusion, Hartgerink et al. (2015) reported that strong effect sizes can be expected in relation to the most immediate outcome measures (i.e., reflexive responses; *Cohen’s d* = 1.36). Since our planned analyses comprised both physiological and self-report measures as a broad category of reflexive responses to two different forms of exclusion (and not acceptance as is commonly the case), we pre-estimated a smaller but still moderate effect size between conditions within the range of *d* = 0.5–0.7. To achieve an alpha value of0.05 and a power value of0.8 for the main planned analyses, we input sample size values within the range of *n* = 65–80. A sensitivity test conducted in G^∗^Power (Erdfelder et al., 1996) suggested a range of estimated effect sizes that fell within the range of our estimations as above. As such, target sample size was preset at *n* = 80, allowing for further exclusions if necessary. Finally, individual *p* values and confidence intervals are reported in the secondary analyses such that no mathematical correction was made for multiple comparisons that are complementary to the main hypothesis testing (Rothman, 1990) and serve only to contextualize the results.

### Sample Demographics

The final sample of eligible participants included in the present study was 80 adults ranging in age from 18 to 40 years old (*M* = 27.05, *SD* = 5.9). There were slightly more females than males (61% female, 39% male) and just under half the sample were students (*n* = 39, 49.3%), five of them were psychology students recruited through the research participation pool at the University of Oslo (6%). 28 were full-time workers (35.4%) and eight were part-time workers (10.1%). The remaining five participants were unemployed or did not answer (5.2%). The sample was highly educated with 55.6% of participants having achieved a bachelor’s degree or higher and the remaining 44.4% having completed high school and at least one year of higher education. A total of 34 participants were single (43%), and the rest were in a relationship or married (57%).

### Pre-Screen Survey

#### Self-Report Measures

All self-report measures included in the online survey used to pre-screen and recruit participants for the laboratory study were forward-back translated from English into Norwegian by a team of bilingual researchers at the University of Oslo, and most were plotted on 5-point visual analog scales (VAS) (unless otherwise stated). We used subscales from the short-form version of Five Factor Narcissism Inventory (FFNI – Vulnerable Narcissism and Indifference; Sherman et al., 2015; Prendergast et al., 2019) and the Experiences in Close Relationship short-form scale (ECR-N; Olssøn et al., 2010) to examine the effect of these individual traits on responses to both forms of exclusion. The Everyday Discrimination Scale (EDS; Williams et al., 1997), the UCLA Loneliness Scale-3 (Hughes et al., 2004), and a newly developed measure of experiences of rejection were used to investigate previous experiences of social exclusion and their impact on reflexive responses in the current study. Finally, the Interpersonal Reactivity Index (IRI; Davis, 1980; Keaton, 2017) was included as a measure to control for user *presence* through its relevant sub-factors *fantasy* and *perspective taking*, both of which are applicable in the case of IVET using an Oculus Rift VR headset.

#### Topic Categorization

Since Blackhart et al. (2009) also included individuals’ “desire to join the group” as a key factor of responses to exclusion, we aimed to control for this feature by recording participants’ views on a topical issue prior to the experiment, that were either shared or opposed by the group from which they would subsequently be explicitly or implicitly excluded during the experimental manipulation. As such, we tried to model their “true” fit or belonging in the group, as a function of shared views on a topical issue. Participants were thus asked to indicate their level of agreement with five statements linked to the content of the discussion that took place among the actors in the IVE. These items were intended to measure participants’ views concerning a topical issue in Norway; maternity leave and traditional motherly roles vs. career development for women (e.g., “*A woman’s most important role in life is motherhood,” “Women should be able to advance in their careers without worrying about playing a motherly role to their children*,” etc.), and, thus, their level of agreement with the actors’ viewpoints, as expressed during the discussion in the experimental manipulation. A mean score across all five items was used to categorize participants into three groups: *progressive*, *neutral*, and *traditional* (see Supplementary Material for more details).

### Equipment

The 360° videos were pre-recorded with a group of professional actors using a Samsung Gear 360 camera that had been set to record on a microphone stand at average height level in front of the group. The videos were stitched and rendered for playback and the camera tripod was masked using Final Cut Pro X (Apple Inc., 2018). The participant wore an Oculus Rift VR headset to experience the social group and eventual manipulation of exclusion. The 360° videos were displayed on the Oculus Rift VR headset by compiling an executable file in Unreal Engine (Epic Games Inc., 2018) to display the videos in the headset without showing any of the user control interface to the participant. The headset was placed over the participant’s head and adjusted accordingly. This headset has an OLED panel for each eye, with a resolution of 1,080 × 1,200. Headphones are integrated in the headset and were placed directly over the participant’s ears during the experiment for audio playback.

We used a BIOPAC MP150 to measure EDA with disposable EL507 electrodes and PPG to extract and compute PR and PRV.

### Experimental Laboratory Study

Upon arrival, participants were first introduced to the laboratory, equipment and procedures. The experiment took approximately 30–45 min per participant.

#### EDA and PPG

In line with recommendations for measuring EDA and PPG, the confirmation email sent to participants had requested that they not consume any caffeinated substances up to a minimum of three hours before the experiment. Before the experiment started, a research assistant first cleaned the participant’s fingers with water and dried with tissue paper. The EL507 electrodes and the PPG sensor were next applied to the participant’s non-dominant hand and this arm was rested on a high table to keep it completely still throughout the experiment. We next ensured a good signal was being recorded while the participant stood with their arm resting on the high table for approximately 3 min.

#### Immersive Virtual Environments

We then placed the Oculus Rift VR headset on the participant. To establish a baseline recording for the physiological measures, we first put participants into a pre-recorded 360° IVE of the empty lab room where they were currently standing. This also functioned as a method to increase *presence* in the IVE for participants (Smolentsev et al., 2017) and lasted 3 min in total. The Oculus Rift VR headset was then removed and participants were asked to carefully read the instruction sheet intended to prepare them for the experimental manipulation.

#### Experimental Instructions

The instruction sheet provided participants with a backstory for the social group they would encounter in the subsequent IVE. The sheet asked participants to imagine that it was their first day working at a new creative company. During the 11 am coffee break, they go to the staff canteen and notice a group of influential employees gathered around a table. We offered participants a motivation to join the group. Half of the participants were randomly given an intrinsic motivation to join the group (i.e., try to make a good impression in this group of new colleagues), while the other half was provided with an extrinsic motivation (i.e., try to make a good impression in this group of new colleagues so as to increase the chances of a promotion and eventual pay rise within the company). See Supplementary Material for more details.

#### Experimental Manipulation

Participants were randomly but evenly assigned to either explicit or implicit exclusion conditions before arriving at the laboratory. The Oculus Rift VR headset was once again fitted onto the participant. They next experienced a 60 s IVE of the empty workplace canteen, as described in the instruction sheet. This enabled a baseline measure of the physiological responses while participants became familiar with the canteen, so that details of the space were not distracting during the subsequent manipulation of exclusion. The Oculus Rift VR headset screen then faded to black for 10 s, and after this time elapsed, faded from black into the experimental manipulation with the social group in the canteen.

The social group and experimental manipulation of exclusion was experienced through the first-person perspective of the participant. It lasted 60 s in total and involved a group of six professional Norwegian actors (four male, two female) who were discussing a colleague’s choice not to return to work after her maternity leave had ended. The discussion lasted 30 s in total in both the explicit and implicit exclusion scenes and four members of the group expressed opinions that collectively affirmed the group’s unified viewpoint on the subject (i.e., either in favor of their colleague’s decision or opposed to it).

In the explicit condition, the first of three overt exclusionary cues happened at 34 s (“*What are you doing here?*”), the second at 37 s (“*We are kinda busy here. There are some free seats over there*”) and the third and final utterance at 44 s (“*You can maybe go now*”). These were verbalized by one male and one female member of the group in an overt and active way and directed at the camera/participant. During the final 14 s of the explicit manipulation, the group acted congruently with the exclusion that had just taken place, such that an awkward silence followed while they smirked or looked disapprovingly at the camera/participant. In the implicit condition, once the group discussion had ended, the group remained silent and simply ignored the participant. All other factors were held constant across conditions.

Finally, there were two content conditions designed to account for any potential differences between *progressive* and *traditional* groups that further enhanced the experimental control of the manipulations. Participants were randomly but evenly assigned to one content condition. In one set of videos, the group espoused more *progressive* views as a unified group and in the second set, they expressed more *traditional* views on the topic. All statements made by each group were matched temporally and semantically in that they were antithetical to one another across conditions. These stimuli did not lead to significant differences across groups so this factor was not included in the final analyses (see Supplementary Material for more details).

#### Positive and Negative Affect Scale (PANAS)

Once the experimental manipulation had concluded, the Oculus Rift VR headset was removed from the participant and they were immediately presented with a smartphone to fill out a short-form version of PANAS, comprising eight semantic differentials of positive and negative emotions plotted on a VAS across.

### Post-experiment Survey

The BIOPAC censor was then removed. Next, participants were asked to fill out some final self-report measures including some manipulation checks and other relevant control variables.

#### Manipulation Checks and Control Variables

Participants answered single item measures about their level of *immersion* in the IVE plotted on a 11-point VAS, labeled from 0% (*not at all*) to 100% (*to an extreme extent*) and about their *perceived level of exclusion or inclusion* in the social group, plotted on a 20-point VAS −10 (*I felt excluded*) to 10 (*I felt included*) (reverse coded for analysis). Other measures included that do not feature in the analyses herein are presented in the Supplementary Material.

## Results

### Analysis

#### Experimental Design

One factor was used to model affective responses to social exclusion (i.e., the explicit or implicit form of the exclusion). We further explore the role of other explanatory variables in contextualizing results in a later section of the article.

#### Manipulation Checks and Control Variables

##### Immersion in the IVE

Participants registered a satisfactory level of immersion in both conditions the IVE (*M*_*Explicit*_ = 56.02%, *SD* = 3.88, *M*_*Implicit*_ = 52.92%, *SD* = 3.94). Relationships between the IRI dimensions of *fantasy* and *perspective taking* and level of immersion in the IVE differed as a function of the form of exclusion experienced, such that *fantasy* and *immersion* were only significantly positively correlated in the explicit exclusion condition [*r* = 0.36, 95% CI (0.06, 0.60), *N* = 41, *p* = 0.02], while *perspective taking* was only positively correlated with *immersion* in the implicit condition [*r* = 0.45, 95% CI (0.16, 0.68), *N* = 39, *p* = 0.003].

##### Perceived level of exclusion or inclusion

Individuals who experienced explicit exclusion (*M*_*Explicit*_ = 8.86, *SD* = 0.35) rated themselves as feeling significantly more excluded from the group than those who experienced implicit exclusion (*M*_*Implicit*_ = 3.32, *SD* = 0.60), (Exclusion_*Explicit*_ − Exclusion_*Implicit*_) = 5.54, 95% CI [4.16, 6.91], *p* = 0.001. The implicit exclusion condition was still experienced as a form of exclusion since the mean response was above 0, where 0 was neutral on the bipolar differential of −10 (extremely included) to +10 (extremely excluded), indicating that participants on average felt excluded by the group in both conditions according to the VAS labels.

#### Effect of Form of Exclusion on Reflexive Responses

##### Positive and negative affect scale

**FIGURE 2:**
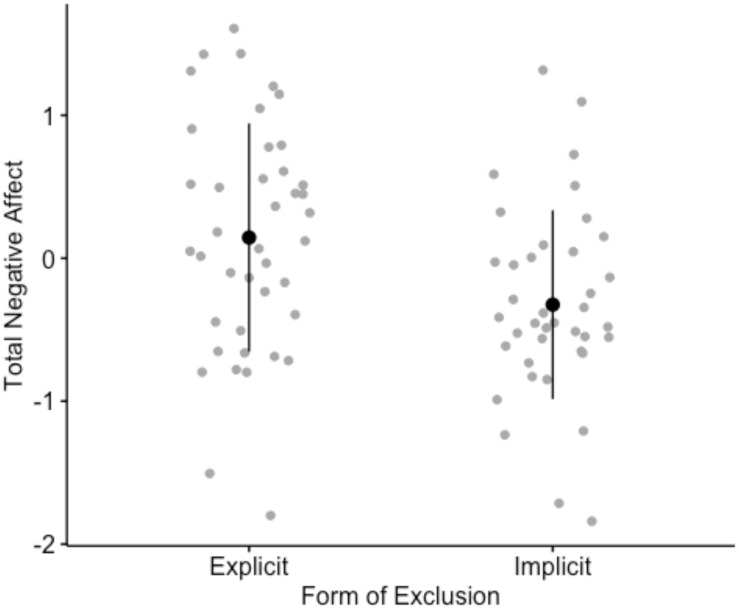
Plots showing the mean, spread and confidence intervals for negative affect across conditions ranging from values –2 to 2.

Results from the self-report PANAS recorded directly after the experimental manipulation exhibited a statistically significant difference between those in the explicit exclusion condition (*M*_*Explicit*_ = 0.14, *SD* = 0.12) and those in the implicit exclusion condition (*M*_*Implicit*_ = −0.32, *SD* = 0.11), such that more NA was reported by those having experienced explicit exclusion, as predicted (NA_*Explicit*_ − NA_*Implicit*_) = 0.46, 95% CI [0.14, 0.80], *p* = 0.0005. However, it is interesting to note that the summed mean reporting across all eight items from the original scale corresponds to a somewhat neutral position on the bipolar differential of positive to NA in both conditions and was only significantly different from zero in the implicit condition (*p* = 0.01) with slightly more positive affect being reported, as shown in Figure 2 (see Supplementary Material for an individual breakdown of affect items).

##### EDA

The data was first filtered and processed using automated analysis routines in Acqknowledge software (Biopac Systems Inc., 2018) and in line with recommendations set out by Boucsein (2012). Data from 13 participants was omitted from the analyses of EDA (*n* = 67). Eight exclusions were due to low quality data with no signal reading, brought about by excessively warm or cold hands. Two participants were excluded due to errors in the synchronization of events with the EDA readings during the recording. A further two participants were deemed to be “non-responders” during testing due to flatline signals across all scenes. One final participant was excluded due to reported use of stimulant medication at the time of testing.

Changes in the tonic signal or SCL that occurred during the experimental manipulation were analyzed with respect to two baseline levels for each individual. The first was a baseline for the experience of an IVE in the Oculus Rift (i.e., the 120 s period during which the participant was placed into the empty lab where they were currently standing, referred to as “lab”), and the second for the immersive experience of a different room to that in which they were currently standing (i.e., the 60 s period in the empty canteen in which they would eventually encounter the social group, referred to as “empty”). A “lab” baseline measure was missing for seven participants due to a change of electrodes between scenes to achieve a better signal. The 60 s experimental manipulation was carved up into three time-frames; “pre” referring to the conversation that the group were having before the exclusion took place, “during” referring to the exclusionary act (explicit or implicit) and “post” referring to the time remaining after the exclusion had taken place and up until the scene ended. For this analysis, the timeframes “during” and “post” were compared to both baseline measures “lab” and “empty” as a difference calculation in SCL across experimental conditions.

With respect to the timeframe “during” exclusion, the SCL levels of participants who experienced explicit exclusion (*M*_*LabExplicit*_ = 0.50, *SD* = 0.09; *M*_*EmptyExplicit*_ = 0.13, *SD* = 0.04) increased significantly more than those who experienced implicit exclusion (*M*_*LabImplicit*_ = 0.28, *SD* = 0.07; *M*_*EmptyImplicit*_ = -0.07, *SD* = 0.03). Differences from the “lab” baseline were significant for explicit exclusion when compared to implicit exclusion (SCL_*Lab*__*Explicit*_ − SCL_*Lab*__*Implicit*_) = 0.23, 95% CI [0, 0.45], *p* = 0.05 and differences from the “empty” baseline across conditions were also significant (SCL_*Empty*__*Explicit*_ − SCL_*Empty*__*Implicit*_) = 0.15, 95% CI [0.05, 0.25], *p* = 0.004.

This same pattern followed for the period “post” exclusion from the baseline measures “lab” and “empty” in the explicit condition (*M*_*LabExplicit*_ = 0.47, *SD* = 0.08; *M*_*EmptyExplicit*_ = 0.09, *SD* = 0.04) when compared to the implicit condition (*M*_*LabImplicit*_ = 0.26, *SD* = 0.08; *M*_*EmptyImplicit*_ = −0.03, *SD* = 0.04), though there was only a significant increase from the “empty” baseline (SCL_*EmptyExplicit*_ − SCL_*EmptyImplicit*_) = 0.13, 95% CI [0.01, 0.24], *p* = 0.02 with a difference below significance across conditions when compared to the “lab” baseline (SCL_*LabExplicit*_ − SCL_*LabImplicit*_) = 0.21, 95% CI [−0.02, 0.43], *p* = 0.075.

We next investigated the amplitude and frequencies of SCRs. Since these were event-related skin conductance responses (ER-SCRs) in the explicit condition due to the explicit utterances but non-specific skin conductance responses (NS-SCRs) in the implicit condition, we compared mean amplitudes across all responses occurring within the timeframes “during” and “post” of the manipulation. While the mean amplitudes in general were higher in the explicit condition (shown in Table 1), these were not statistically different across experimental conditions.

**TABLE 1:** Mean amplitudes “during” and “post” exclusion segments across conditions.

Segment	Condition	*n*	*M*	*SD*	[95% CI]
During exclusion	Explicit	24	0.275	0.052	[0.171, 0.379]
	Implicit	24	0.165	0.027	[0.111, 0.218]
Post exclusion	Explicit	14	0.204	0.032	[0.139, 0.269]
	Implicit	24	0.15	0.036	[0.077, 0.224]

The amplitude of the first SCR occurring “during” the exclusion in the explicit condition (*M*_*Explicit*_ = 0.29, *SD* = 0.06) and the implicit condition (*M*_*Implicit*_ = 0.17, *SD* = 0.03), was higher in the explicit condition, but just below statistical significance (AMP1_*Explicit*_ − AMP1_*Implicit*_) = 0.12, 95% CI [−0.01, 0.24], *p* = 0.07. There were no differences between the amplitudes of subsequent SCRs across conditions.

##### HR and HRV

In our analyses, we used root-mean square differences of successive RR intervals (RMSSD) as an index for HRV and beats per minute (BPM) for HR, calculated at one-minute intervals. Both were extracted and computed using automated analysis routines in Acqknowledge software (Biopac Systems Inc., 2018). Data from seven participants was omitted from HR and HRV analyses (*n* = 73). Four exclusions were due to extremely low-quality data with significant artifacts present in the PPG signal due to physical movements of the participant. Two participants were excluded due to errors in the synchronization of stimulus presentation and the PPG recording. A final participant was excluded due to reported use of stimulant medication at the time of testing. Additional RMSSD values were coded as missing for particular segments due to unreliability (i.e., above the acceptable range of RMSSD values for the sample age and gender (van den Berg et al., 2018), that had been derived erroneously from artifacts in the PPG signal and were detected manually.

As before, changes in the HRV that occurred “during” and “post” exclusion while in the IVE were analyzed with respect to two baseline levels for each individual from the “lab” and “empty” IVEs. The HRV of participants who experienced explicit exclusion (*M*_*DuringExplicit*_ = −24.55, *SD* = 4.69; *M*_*PostExplicit*_ = −24.47, *SD* = 5.09) decreased significantly more when compared with those in the implicit condition (*M*_*DuringImplicit*_ = −11.18, *SD* = 4.00; *M*_*PostImplicit*_ = −8.91, *SD* = 6.09) with respect to the “empty” baseline when compared with “during” and “post” exclusion segments (HRV_*DuringExplicit*_ − HRV_*DuringImplicit*_) = −13.36, 95% CI [−25.66, −1.07], *p* = 0.03 and (HRV_*Post*__*Explicit*_ − HRV_*Post*__*Implicit*_) = −15.56, 95% CI [−31.4, 0.28], *p* = 0.05. When comparing the RMMSD changes that occurred “during” and “post” exclusion segments across conditions, we found that explicit exclusion engendered ongoing decreases while implicit exclusion led to small increases or recoveries to baseline HRV, though not to a statistically significant degree.

Finally, our analyses of HR were inconclusive. We used mean BPM during the exclusion segments “during” and “post” to measure changes from both baseline mean BPM values and consecutively during the experimental manipulation. On average, mean BPM remained stable during the experimental manipulation with a general tendency toward small decreases overall.

#### Correlational Analyses and Contextualization of Results

With respect to our EDA results, when we explored the correlations between the amplitudes (qualitative response) and frequencies (quantitative response) of specific SCRs “during” and “post” exclusion, we found that the individual difference variables that we had measured correlated significantly with these amplitudes in the implicit condition but not in the explicit condition. Moderate to strong positive correlations were found between individual amplitudes exhibited in the “post” exclusion phase with FFNI vulnerable narcissism [*r* = 0.45, 95% CI (0.07, 0.73), *N* = 24, *p* = 0.02], introspection [*r* = 0.41, 95% CI (0.01, 0.70), *N* = 24, *p* = 0.04), anxious [*r* = 0.43, 95% CI (0.04, 0.71), *N* = 24, *p* = 0.03], and avoidant [*r* = 0.87, 95% CI (0.23, 0.98), *N* = 24, *p* = 0.02] attachment, although confidence intervals were long. The “post” exclusion phase is precisely when individual differences across relational variables come into play qualitatively in processing the event. However, and as hypothesized, this is strongest when implicit exclusion is cued. The mean amplitude of SCRs recorded across the entire “post” exclusion correlated positively with FFNI vulnerable narcissism [*r* = 0.34, 95% CI (0, 0.61), *N* = 34, *p* = 0.05] in the implicit condition, again with a relatively long confidence interval. Conversely, in the explicit condition, the overall mean amplitude of SCRs exhibited in the “post” exclusion phase had a negative correlation with FFNI vulnerable narcissism [*r* = −0.56, 95% CI (−0.84, −0.05), *N* = 14, *p* = 0.04]. This suggests that a higher level of vulnerable narcissism buffers against the negative effects of explicit exclusion. The only significant correlation found between the overall mean amplitude exhibited across both “during” and “post” exclusion frames by those in the explicit condition was a moderate negative correlation with UCLA loneliness [*r* = −0.39, 95% CI (−0.67, −0.01), *N* = 27, *p* = 0.04], suggestive that increased feelings of loneliness in general may buffer against the effect of the explicit exclusion.

The frequency of SCRs “during” and “post” exclusion, as a quantitative measure, were also on average the same across explicit and implicit exclusion conditions. However, similarly, when we looked at the relationships between some of the relational variables and the frequencies of SCRs exhibited “during” implicit exclusion, we found significant moderate negative correlations with the pre-recorded measures of prior experiences of rejection [*r* = −0.42, 95% CI (0.66, −0.10), *N* = 36, *p* = 0.01] and everyday discrimination [*r* = −0.5, 95% CI (0.73, −0.25), *N* = 36, *p* < 0.001], FFNI vulnerable narcissism [*r* = −0.44, 95% CI (−0.67, −0.13), *N* = 36, *p* = 0.007] and a positive correlation with the item measuring perceived level of exclusion subsequent to the manipulation [*r* = 0.37, 95% CI (0.05, 0.62), *N* = 36, *p* = 0.03]. There were no significant correlations between any control variables and the frequency of SCRs exhibited by those in the explicit condition, suggestive that it was the explicit form of exclusion alone that engendered the frequency of SCRs in the explicit condition. Whereas in the implicit condition, the frequencies were significantly and quantitatively linked to the relational experiences and traits that we had previously measured.

Concerning HRV results, there were no meaningful associations between relational variables and changes in HRV “during” exclusion in the explicit condition. In the implicit condition, however, decreases in HRV were significantly associated with higher levels of the IRI trait dimension *personal distress* [*r* = −0.37, 95% CI (−0.63, −0.06), *N* = 36, *p* = 0.02] and anxious attachment [*r* = −0.33, 95% CI (−0.60, −0.004), *N* = 36, *p* = 0.05].

Again, when contextualizing and interpreting the results with respect to the different forms of exclusion experienced, those who registered higher levels of perceived exclusion in the implicit condition had also reported lower levels of loneliness [*r* = −0.34, 95% CI (−0.59, −0.02), *N* = 39, *p* = 0.04], fewer prior experiences of rejection [*r* = −0.34, 95% CI (−0.59, −0.02), *N* = 39, *p* = 0.04], and discrimination [*r* = −0.37, 95% CI (−0.61, −0.07), *N* = 39, *p* = 0.02] in the pre-screen survey. These relationships were not present in the data of those who were explicitly excluded during the experiment. In addition, there were significant main effects, but no interaction effects, in a multiple regression of the form of exclusion (i.e., implicit exclusion) on these three related variables when modeled separately as independent variables on the perceived level of exclusion as shown in Table 2.

**TABLE 2:** Regression analyses with perceived level of exclusion as criterion.

Predictor	β	95% confidence interval	*a*	*R*^2^
UCLA loneliness	−0.146	[−0.31, 0.02]		
Implicit exclusion	−1.294**	[−1.62, −0.96]	0.632	0.47**
Experiences of rejection	−0.194*	[−0.36, −0.03]		
Implicit exclusion	−1.345**	[−1.67, −1.02]	0.645	0.49**
Everyday discrimination	−0.204*	[−0.39, −0.02]		
Implicit exclusion	−1.310**	[−1.63, −0.99]	0.618	0.49**

*Indicates p < 0.05 and **indicates p < 0.01.

## Discussion

In this present study, participants experienced either explicit or implicit social exclusion in an IVE with real actors. Their reflexive responses were recorded with respect to self-report positive and NA directly after the exclusion and electrodermal and cardiovascular activity during the manipulation. Our results show that explicit exclusion generates higher levels of NA than implicit exclusion. Implicit exclusion elicits stress responses almost on a par with explicit exclusion, but in the case of implicit exclusion, these responses are correlated with other relational variables and experiences and not the exclusionary event in and of itself, as is the case with explicit forms.

Our main findings are as follows. Individuals in both the explicit and implicit condition reported that they felt excluded from the social group they encountered in the IVE, but to a more extreme degree in the explicit condition, as expected. Participants also reported satisfactory levels of *immersion* in the scene and thus *behavioral realism* was achieved. User *presence* was also sufficient but had different functions, with the impact of explicit exclusion borrowing more from *fantasy* and the effect of implicit inclusion relying more on *perspective taking*. Taken together, the novel exclusion paradigm we used in this experiment passed the manipulation tests and control parameters set out to ensure its ecological validity using IVET. Given that the scene itself was immersive and individuals reported feeling some level of exclusion from the group, we could be confident that we had worked toward our methodological aim of increasing the ecological validity and experimental control of the manipulation.

There were significant differences in reflexive responses to explicit and implicit forms of exclusion. Crucially, the experience of explicit exclusion generated more NA than implicit exclusion, yet both could be interpreted as relative states of “emotional numbness” when mean values on the self-report PANAS were investigated, since they fell around 0 on the bipolar VAS of positive (−3) to negative (+3) affect, where 0 indicated neutrality. The use of a bi-polar VAS and the spread of results across conditions sheds some light on the interpretation of findings that may otherwise have blanketed one another within and across studies assessing affective responses to exclusion as a general phenomenon. In relation to electrodermal and cardiovascular responses, explicit exclusion led to even higher physiological arousal than implicit exclusion, indexed by higher SCLs “during” and “post” exclusion and lower HRV “during” exclusion. Both experimental conditions led to physiological arousal when compared to baselines, generating increases in EDA, as well as decreases in HRV, consistent with previous reports on reactions to stressful situations such that we could be confident both conditions had the intended effect on participants and physiological arousal is indeed detectable in conditions of exclusion. However, we did find that increases in HR seemed to remain stable across condition segments and the conditions in general. This could be an indication that changes in HR happen more rapidly and are better compared across shorter periods of time (i.e., intervals less than 60 s). Given the differences in physiological responses that were clearly present in our other measures, HRV may be a better general measure than HR in accounting for changes in cardiovascular activity that occur during exclusionary events.

In the contextualization of results, the reflexive responses we recorded also correlated with the previously recorded self-report measures relating to individual traits and prior experiences of exclusion, but only in the implicit condition. These results are presented as secondary analyses and helped to interpret the data that, at times, did not differ significantly between exclusion conditions. For example, it appears SCRs are directly triggered by cues of explicit exclusion since they did not relate to other relational factors that we controlled for, whereas individual traits and prior relational experiences were significantly related to SCRs when exclusion was implicitly cued. While both conditions generated similar amplitudes and frequencies of SCRs, importantly, these had inverse relationships with the control variables across conditions. While we didn’t find strong statistical interactions in this respect, these correlations begin to provide evidence that some of the relational variables we included may contribute to the intensity of the experience of implicit exclusion, but also buffer against prolonged negative feelings once exclusionary events have concluded. As well as being evident in some of the EDA data, the buffering effect of ongoing experiences of loneliness in life, everyday experiences of rejection and discrimination, was also present at the perceived level of exclusion of those who had experienced implicit exclusion. We understand this pattern to be in line with our theory that responses to implicit exclusion hinge more generally on prior and ongoing relational experiences that signal and underpin more implicit forms of social exclusion in everyday life. However, the data give a first indication that they may actually serve as buffers, rather than aggravators, in generating reflexive responses where exclusion is more implicitly cued.

## Conclusion

In sum, the present study was successful in implementing a novel exclusion manipulation of two different forms of exclusion using IVET in a laboratory setting. As this technology advances and becomes easier to acquire, we may continue to increase the ecological validity and experimental control of laboratory experiments using IVEs. In addition to the increased experimental control afforded by the new exclusion paradigm we developed, and the strict experimental protocol that we followed, the sample population was also more varied than most laboratory studies given the range of values recorded across population demographics.

Futhermore, the results of the PANAS self-report measure present a possible explanation for the inconsistencies found in the exclusion literature, as outlined by Blackhart et al. (2009), where both high levels of NA and “emotional numbness” have been reported. Without a distinction between the form of exclusion being made, it is possible that the effects have blanketed one another within and across previous research leading to inconclusive metanalyses. Therefore, the results of the current study highlight the importance of understanding how the form of exclusion experienced can have a differential impact on affective responses, helping to better predict the different patterns that, before now, appeared to run contradictory to one another. Since we also showed that physiological arousal was present at the level of electrodermal and cardiovascular activity in conditions of both explicit and implicit exclusion, this helped to address prior questions as to whether social exclusion impacts physiology in a detectable way. We found that HRV may be a more suitable candidate than HR in understanding cardiovascular responses.

Finally, our secondary analyses further helped to contextualize the reflexive responses recorded in terms of prior relational experiences and individual differences when their effects on other dependent variables were non-significant between conditions of exclusion. These analyses can begin to inform future work about how reflexive responses to implicit exclusion, in particular, are linked to individual difference factors, while explicit exclusion alone can lead to increased NA and physiological arousal without necessarily calling these relational factors into play.

## Data Availability Statement

The datasets presented in this study can be found in online repositories. The names of the repository/repositories and accession number(s) can be found below: https://osf.io/5d8jc/?view_only=9a9909927f8f4d489df4ba04abe4d9b3.

## Ethics Statement

The study content and data management protocol were approved by the national data protection service in Norway and the Institutional Review Board. Participants gave their explicit consent for participation in the pre-screen survey online by signing their names in a consent box and provided their details to be contacted for participation in the laboratory. Written informed consent was obtained from the actors for the publication of any potentially identifiable images or data included in this article.

## Author Contributions

CP designed the study and created study materials, collected and analyzed the data, and drafted the manuscript. TS advised on data collection, analysis and interpretation of data, and revised the manuscript. All authors contributed to the article and approved the submitted version.

## Conflict of Interest

The authors declare that the research was conducted in the absence of any commercial or financial relationships that could be construed as a potential conflict of interest.
