# Supplementary material for: Investigating Reflexive Responses to Explicit and Implicit Forms of Social Exclusion Using Immersive Virtual Environment Technology
Source: Front Psychol. 2020 Oct 6;11:575783. doi: 10.3389/fpsyg.2020.575783 (PMC7573141; doi:10.3389/fpsyg.2020.575783)
Supplement: Supplementary file 1 [file Table_1.DOCX]

**Materials and Methods**

**Self-Report Survey Measures**

***Pre-Screen Survey***

Various self-report measures were included in a pre-screen survey used to recruit participants.

**Five Factor Narcissism Inventory (FFNI).** The long-form version of the FFNI consists of 148 self-report items [(Glover, Miller, Lynam, Crego, & Widiger, 2012)](https://www.zotero.org/google-docs/?O3R1Ac). In the current study, we included the short-form version for FFNI Vulnerable Narcissism, encompassing the four subscales of Reactive Anger; Shame; Need for Admiration; and Distrust/Cynicism and one sub-dimension of FFNI Grandiose Narcissism Indifference, each composed of 4 items per subscale [(Prendergast et al., 2019; Sherman et al., 2015)](https://www.zotero.org/google-docs/?vexl9d).

**Experiences in Close Relationships (ECR)**. ECR is a measure of anxious and avoidant attachment styles [(Brennan, Clark, & Shaver, 1998)](https://www.zotero.org/google-docs/?L2HOhp). A previously validated Norwegian translation, ECR-N [(Olssøn et al., 2010)](https://www.zotero.org/google-docs/?p38Yl1), was included. It is a short-form 12-item measure that has been shown to have satisfactory psychometric properties.

**Introspectiveness**. This scale is an 11-item measure of the extent to which one thinks about and processes one’s inner feelings and thoughts [(Hansell & Mechanic, 1985)](https://www.zotero.org/google-docs/?eNUnKP). It was included in the pre-screen survey as a control variable for one of the distractor measures relating to a separate student project.

**Interpersonal Reactivity Index (IRI).** IRI is a 28-item scale with four subscales; Empathic Concern*,* Personal Distress, Perspective Taking and Fantasy. IRI was developed to capture individual differences in cognitive and emotional reactions to different interpersonal events and stimuli [(Davis, 1980; Keaton, 2017)](https://www.zotero.org/google-docs/?KwOwnK). The dimensions of Perspective Taking and Fantasy were included as control variables for the effectiveness of the experimental manipulation in the laboratory study.

**The Everyday Discrimination Scale (EDS)**. EDS is a 9-item measure of subjective experiences of discrimination [(Williams et al., 1997)](https://www.zotero.org/google-docs/?ysGT8g). It was implemented as a count variable scored on a 5-point Likert scale from 0 (*never*) to 4 (*always*). Since everyday experiences of discrimination are often linked to sustained feelings of marginalization on a broader social level, it was included to control for the effect of the experimental manipulation of exclusion on responses.

**Experiences of Rejection.** This 6-item measure was developed to quantify how frequently individuals experience common forms of rejection and exclusion. Included as a count variable scored on a 5-point Likert scale from 0 (*never*) to 4 (*always*), the items are summed together to give an overall subjective measure of the frequency of experiences of exclusion. Items include: *I am excluded from conversations in groups*; *I am not invited to social events by people I know*; *At social events, people I know choose to sit with others instead of with me; I get blanked on the street by people I know; People ignore my calls/messages; I am left out of group activities.* Frequent or prolonged exposure to these common types of exclusion in everyday life may in turn affect participant responses to exclusion in the experimental setting.

**UCLA Loneliness Scale-3.** UCLA-3 is a short-form of the standard measure of loneliness derived from the R-UCLA loneliness scale. This scale was shortened for inclusion in large-scale social surveys and consists of 3 questions to measure experiences of loneliness in a broad and general way [(Hughes et al., 2004)](https://www.zotero.org/google-docs/?PAMmaO) and is plotted on a 5-point likert scale. Loneliness is often reported as a long-term outcome of ongoing and sustained feelings of exclusion from social groups.

**Meaningfulness and Purpose in Life.** A single item self-report measure was developed to capture the meaningfulness and purpose an individual feels they have in their life; *Do you feel that your life is meaningful and has a purpose?* It was measured on a 20-point visual analogue scale with labels ranging from -10 (*definitely not*) to 10 (*definitely yes*). Embedded in one of the distractor measures, it was included as part of a repeated measures design and presented again to the participant in a self-report survey that followed the experimental manipulation to assess any possible effects that the experience of different types of social exclusion may have on this variable.

**Other measures.** Participants also answered two distractor measures relating to ‘willingness to be teleported’ and ‘unconscious motivations’, that were part of a separate student project, and included so as not to influence participants’ expectations of the content of the upcoming laboratory experiment.

***Post-Study Survey***

**Desirability for Control Scale (DCS).** DCS is a measure of how much control individuals wish to have over external events taking place in their lives [(Burger & Cooper, 1979)](https://www.zotero.org/google-docs/?UDPY0P). A lack of control over external events is of particular salience when individuals are explicitly excluded by social others. This variable is thus modelled as a mediator of responses to explicit or implicit forms of exclusion.

**Social Comparison Scale (SCS).** SCS measures individual’s self-perceptions of social rank and relative social standing [(Allan & Gilbert, 1995)](https://www.zotero.org/google-docs/?hNhvdy). Participants are thus asked to make a global comparison of themselves in relation to other people concerning their rank, attractiveness and how well they ‘fit in’ with others in society. This variable is modelled as a mediator of responses to exclusion.

**Social Safeness and Pleasure Scale (SSPS).** The SSPS is designed to capture individual’s feelings of social warmth and safety [(Gilbert et al., 2009)](https://www.zotero.org/google-docs/?gCrUg5). It asks individuals to express their level of agreement with statements relating to belonging and acceptance in society in general. How participants rate these aspects of social life is modelled as a mediational feature of responses to exclusion.

**Desire to be accepted by the group.** This constituted a one-item measure of whether individuals would *normally* want to be accepted by this kind of group.

**Desire to fit in with the group.** An additional one-item measure of whether participants would *normally* want to fit in with this kind of group.

**Ethical Approval and Consent**

The study content and data management protocol were approved by the national data protection service in Norway and the Institutional Review Board. Participants gave their explicit consent for participation in the pre-screen survey online by signing their names in a consent box and provided their details to be contacted for participation in the laboratory study. Individuals who were invited to take part in the laboratory study were asked to carefully read and sign an informed consent form at the laboratory before participating in the experiment. They were also fully debriefed after the experiment and asked to sign a non-disclosure information sheet. They were presented with a 200 NOK universal gift card at the end of the experiment. All participants were given the opportunity to ask questions or retract their participation during the study, and up to three months after the study had been completed.

**Training & Pilot Study**

The experiment was run over the course of two months by a team of 5 research assistants and one lead experimenter. The research team was trained to give the exact same information to all participants by following a script and to act in the same way throughout the experiment. To arrive at an optimal experimental procedure and protocol, and for the research team to become fully competent in the use of the study equipment, we ran a pilot study with 13 participants across the explicit and implicit exclusion conditions and a third experimental condition of acceptance. The acceptance condition was tested to rule out any physiological responses that occurred as a response to communication that was directed toward the camera/participant by the actors, that was a feature of the explicit exclusion condition, but not the implicit exclusion condition. Given that responses in electrodermal activity of those tested in the acceptance condition did not increase when the actors communicated with the camera/participant directly, as they did in the explicit exclusion condition, we decided to proceed by testing the explicit and implicit exclusion conditions only, as the focus was in comparing the differential effects of these two forms of exclusion.

**Results**

**Desire to Join the Group**

A secondary factor was intended to account for individuals’ ‘desire to join the group’, which was recorded as two subfactors (i.e. their ‘true’ and ‘directed’ motivation to join the group).

**Topic Categorization.**

***True motivation***

For ‘true’ motivation, those who had a mean score between -2 and -.05 on the topic measure were classified as *progressive*, meaning that they favoured the idea of women advancing in their careers over occupying more traditional familial roles. Those who had a mean score between -.05 and .05 were classified as *neutral* and those who had a mean score between .05 and 2 were classified as *traditional* according to their endorsement of statements that placed importance on the mother’s traditional role in the home over career development. The distribution of individuals into these groups was negatively-skewed leaving too few eligible participants in the *traditional* category to balance condition assignment. Therefore, the laboratory study was conducted primarily with those categorised as *progressive* (n=37) and *neutral* (n=39), while just 4 individuals from the *traditional* category took part.

***Directed motivation***

The ‘directed’ extrinsic or intrinsic motivation was presented in the instruction sheet to prepare participants for the experimental manipulation. 39 participants were given an intrinsic motivation to join the group (i.e. participants were instructed to approach the group and try and make a good first impression). The remaining 41 participants were extrinsically motivated and instructed to approach the group and try to make a good impression in order to secure future promotion within the company, that would lead to a pay increase. This factor variable was expected to interact as a function of form.

**Analyses**

We did attempt to extend our analyses to a 2x2 factorial when and where appropriate to show additional effects relating to individuals’ ‘true’ and ‘directed’ desire to join the group on our outcomes variables, but this resulted in as little as 10 participants per cell, and as we did not have access to previous research to adequately calculate power for this factor, we assumed that the lack of significant differences in the interaction of this factor with form on our dependent variables was mainly a reflection of it being underpowered.

Otherwise, it is also possible that the ‘true’ motivation, as modelled in the study, did not adequately distinguish between individuals’ responses as roughly half the sample (i.e. 39 participants) were categorized as *neutral* on the topic. Therefore, we had no clear unbiased measurement of their endorsement of the views expressed by the actors in either content condition (i.e. *progressive* or *traditional*) and as such, no indication of their ‘true’ motivation to affiliate with the social group. Given their neutrality on the topic of conversation within the group, these participants may have judged their fit in the group according to other parameters that we did not control for. Moreover, there were no differences between those who experienced exclusion in the *progressive* and *traditional* content conditions in any of the results. To engage participants in the experiment, all had been instructed to try to make a good impression in the group, irrespective of their ‘true’ motivation to do so.

The most influence the sub factor of ‘directed’ motivation had in our data was for those who experienced explicit exclusion who reported even higher negative affect if given an extrinsic motivation than those who had been given an intrinsic motivation to affiliate with the group, but just below significance (Explicit Extrinsic - Explicit Intrinsic) = .44, 95% CI [-.21,1.10], p = .18.

**Thematic Control Variables**

We also included some thematic control variables in the last part of the study, such as a one-item measure of agreement with the actor’s statements from their conversation in the IVE. However, we believe that these measures were influenced by the exclusion manipulation itself, and in particular, by the explicit exclusion manipulation. A strong indication of this influence on answers was that this variable did not correlate with the pre-screen measure of support for the topic measured prior to laboratory experiment in either the progressive or traditional direction. However, in the implicit condition, prior support for the topic was strongly negatively correlated with the other thematic control variables that were measured, desire to fit in with the group (-.63) and desire to be accepted by the group (-.65), but only if participants were in a group that accorded with their views, i.e. those classified as ‘progressive’ who were assigned to the *progressive* condition.

Participants also indicated if they would *normally* want to be *accepted* by this kind of social group and if they would *normally* want *to fit in and belong to* this kind of group. Analyses of this aspect of the research will be presented in a separate article.

**Positive and Negative Affect Scale (PANAS)**

A breakdown of items featuring on the PANAS is given in Table 1. Only results on two items (*upset* and *annoyed*) were significantly different across groups, while the remaining items were just below significance *p =* .076 - .183, or in the case of *insecure* or *ashamed*, almost identical. Against our prediction that there would be more *fear* than *anxiety* reported in the explicit condition than in the implicit and vice versa, there were no main differences between reporting on either affect across conditions. It is interesting that the effects were stronger for transient emotions that relate to the situation and did not seem to have an effect on personal trait emotions such as confidence and pride. Future research should explore these bipolar dimensions of affect more in the context of exclusion research.

| **Table 1** |  |  |  |  |
| --- | --- | --- | --- | --- |
| *Means of individual Items on PANAS* |  |  |  |  |
| Negative Affect | *Condition* | *M* | *SD* | *[95% CI]* |
| Fear |  |  |  |  |
|  | Explicit | 0.12 | 0.27 | [-0.41, 0.66] |
|  | Implicit | -0.43 | 0.24 | [-0.91, 0.05] |
| Anxiety |  |  |  |  |
|  | Explicit | -0.11 | 0.25 | [-0.61,0.39] |
|  | Implicit | -0.66 | 0.20 | [-1.06,-0.26] |
| Upset** |  |  |  |  |
|  | Explicit | 0.70 | 0.19 | [0.31,1.09] |
|  | Implicit | -0.23 | 0.20 | [-0.63, 0.17] |
| Annoyed** |  |  |  |  |
|  | Explicit | -0.06 | 0.22 | [-0.48, 0.37] |
|  | Implicit | -0.84 | 0.22 | [-1.28, -0.40] |
| Apathetic |  |  |  |  |
|  | Explicit | 0.20 | 0.21 | [-0.21, 0.61] |
|  | Implicit | -0.19 | 0.20 | [-0.59, 0.22] |
| Sad |  |  |  |  |
|  | Explicit | 0.23 | 0.14 | [-0.06, 0.52] |
|  | Implicit | -0.10 | 0.11 | [-0.33, 0.13] |
| Ashamed |  |  |  |  |
|  | Explicit | -0.05 | 0.10 | [-0.26, 0.16] |
|  | Implicit | -0.10 | 0.10 | [-0.36, 0.25] |
| Insecure |  |  |  |  |
|  | Explicit | 0.12 | 0.18 | [-0.23, 0.48] |
|  | Implicit | -0.05 | 0.15 | [-0.36, 0.25] |
| *Note.* ** *p* < 0.01. |  |  |  |  |
